# Effects of armed conflict on child health and development: A systematic review

**DOI:** 10.1371/journal.pone.0210071

**Published:** 2019-01-16

**Authors:** Ayesha Kadir, Sherry Shenoda, Jeffrey Goldhagen

**Affiliations:** 1 Malmö Institute for Studies of Migration, Diversity and Welfare, Malmö University, Malmö, Sweden; 2 Médecins Sans Frontières, Geneva, Switzerland; 3 Division of Community and Societal Pediatrics, University of Florida College of Medicine—Jacksonville, Jacksonville, Florida, United States of America; Aga Khan University, PAKISTAN

## Abstract

**Background:**

Armed conflicts affect more than one in 10 children globally. While there is a large literature on mental health, the effects of armed conflict on children’s physical health and development are not well understood. This systematic review summarizes the current and past knowledge on the effects of armed conflict on child health and development.

**Methods:**

A systematic review was performed with searches in major and regional databases for papers published 1 January 1945 to 25 April 2017. Included studies provided data on physical and/or developmental outcomes associated with armed conflict in children under 18 years. Data were extracted on health outcomes, displacement, social isolation, experience of violence, orphan status, and access to basic needs. The review is registered with PROSPERO: CRD42017036425.

**Findings:**

Among 17,679 publications screened, 155 were eligible for inclusion. Nearly half of the 131 quantitative studies were case reports, chart or registry reviews, and one-third were cross-sectional studies. Additionally, 18 qualitative and 6 mixed-methods studies were included. The papers describe mortality, injuries, illnesses, environmental exposures, limitations in access to health care and education, and the experience of violence, including torture and sexual violence. Studies also described conflict-related social changes affecting child health. The geographical coverage of the literature is limited. Data on the effects of conflict on child development are scarce.

**Interpretation:**

The available data document the pervasive effect of conflict as a form of violence against children and a negative social determinant of child health. There is an urgent need for research on the mechanisms by which conflict affects child health and development and the relationship between physical health, mental health, and social conditions. Particular priority should be given to studies on child development, the long term effects of exposure to conflict, and protective and mitigating factors against the harmful effects of armed conflict on children.

## Introduction

**Fig 1:**
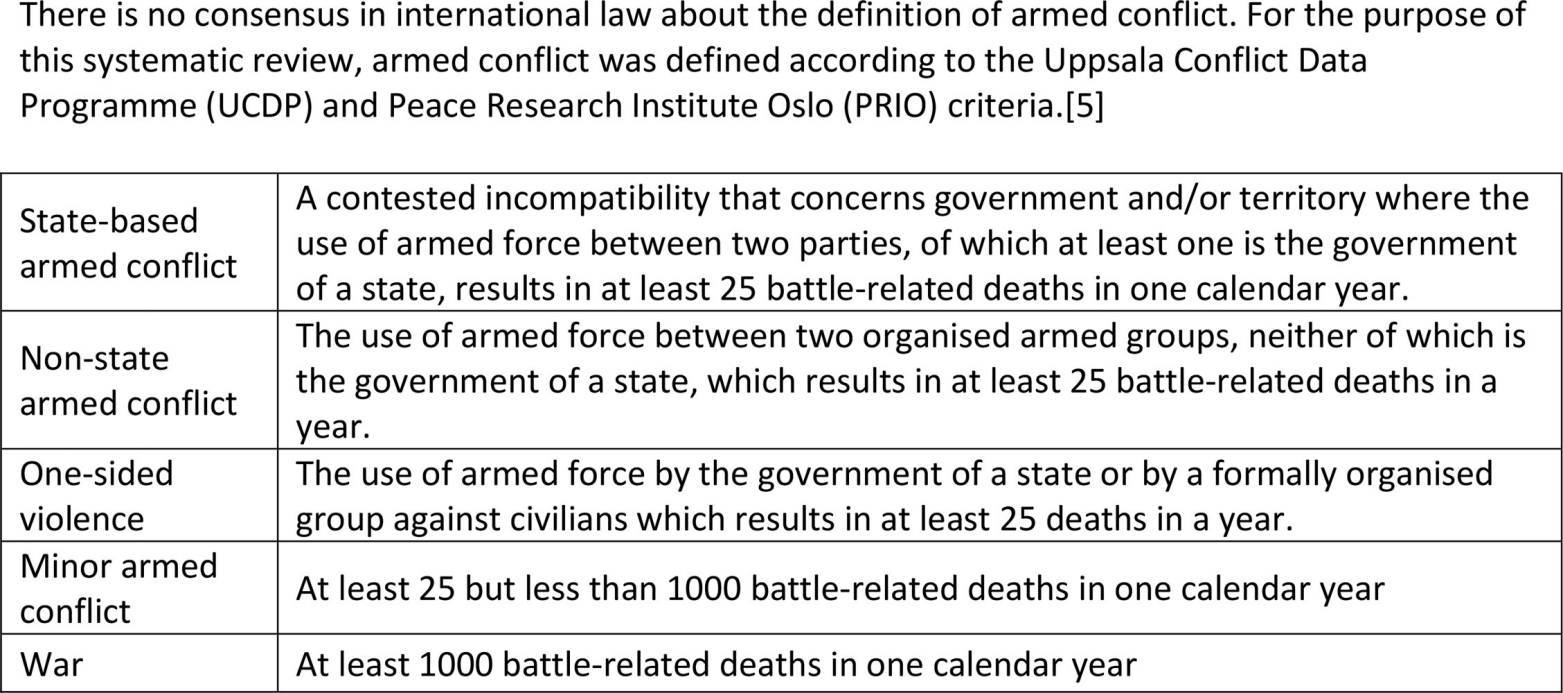
Definitions of armed conflict and conflict intensity.

Millions of children are thought to be impacted by armed conflict worldwide, with estimates for the number of children living in areas affected by conflicts ranging as high as 246 million.[1] Over the past several decades schools, health facilities, and health workers have become direct targets, increasing the impact of war on children.[2, 3] Of the 49 armed conflicts that occurred in 2016, 12 were wars (Fig 1).[4, 5]

Children who are exposed either directly or indirectly to armed conflict suffer harm that persists across their life course and beyond, to subsequent generations born after the conflict has ended. Although this impact has been anecdotally chronicled in news reports and literature, there is limited medical and public health research on how conflict affects on child physical health and development. Even the number of children directly or indirectly affected by conflict remains unclear.

The direct effects of combat on child health may include injury, illness, psychological trauma, and death. A complex set of political, social, economic, and environmental factors resulting from conflicts have indirect and lasting effects on children. Inadequate living conditions, environmental hazards, such as damaged buildings and unexploded ordnance, and lack of access to safe water and sanitation place children at risk for preventable and treatable diseases and injuries. The destruction of medical and public health infrastructure make it difficult to treat affected children by limiting both access and quality of available care.

Conflicts force children and families to leave their homes to seek safety within national borders (internal displacement) and across international borders—nearly two-thirds of the 28 million forcibly displaced children are internally displaced.[6] During flight, children may become separated from their families and are more vulnerable to infections, psychological trauma, and exploitation.[7, 8] Experiences of trauma affect children’s mental health, as well as that of their caregivers. Poor mental health of caregivers may negatively affect children’s physical and mental health, as well as their educational attainment and life opportunities.[8, 9] The destruction of educational and economic infrastructure creates conditions of poverty, which may last for generations. Economic and political sanctions deepen this poverty and have detrimental effects on child health and nutrition.[10]

Little is known about the impact of armed conflict on children’s physical health and development—even estimates of the number of children killed by conflict are lacking.[11–14] Research has focused primarily on the mental health effects of armed conflict on children and on downstream effects such as displacement.[9, 15–20] We undertook a systematic review of the evidence of the impact of armed conflict on children’s physical health and child development. Where available, risk factors, mitigating factors, and protective factors were abstracted.

## Methods

### Search strategy and selection criteria

Searches were undertaken in PubMed, Web of Science, CINAHL, EMBASE, Latin American and Caribbean Health Science (LILACS), IndMED, Africa-Wide Information, Open Grey and the New York Academy of Medicine Grey Literature Report from 1 January 1945 to the search date. The initial searches were performed 8–12 June 2015. The PubMed and EMBASE searches were updated on the 24 and 25 April 2017, respectively. The review is registered with PROSPERO: CRD42017036425.

Our intention was to perform a systematic review and meta-analysis of available data on the physical health and developmental effects of armed conflict on children. During the searches, it became clear that the varied focus, heterogeneous design, and variation in reporting of outcomes by published studies would not support this type of review. The aim of our review was therefore shifted to describe published studies on the effects of armed conflict on child health and development.

Search terms included multiple variants of “child” and “war.” Terms for physical health and child development were not used, as inclusion of these terms narrowed the search results and missed relevant papers known to the authors. The search terms used are provided in the web appendix. Medical Subject Headings terms were used when available, and snowball and hand searching was used to identify additional studies.

Screening and full text review was conducted by Ayesha Kadir (AK) and Sherry Shenoda (SS) for all publications using Covidence,[21] an electronic organisational tool for systematic reviews. Inclusion criteria included study population, setting of past or current armed conflict or a region where refugees/asylum-seekers are staying, and exposure of the study population to armed conflict. Armed conflict was defined according to the Uppsala Conflict Data Programme (UCDP)/Peace Research Institute Oslo (PRIO) criteria (Fig 1).[5] The UCDP database was used to identify conflicts meeting criteria for inclusion.[22] We included original research studies that provided data on children ages 0–18 years. Outcomes included physical or developmental morbidity associated with exposure to armed conflict, exposure to violence, and access to basic needs, including health care and education. Studies on mental and behavioural health were excluded unless they also provided data on physical health or child development. Additional exclusion criteria included review papers, studies published prior to 1945, and studies with a median date of data collection earlier than 1940. Studies on terrorism were excluded, as terrorist incidents are not universally associated with armed conflict. The Palestinian-Israeli conflict was considered to be an armed conflict. Studies providing data exclusively on nutrition, perinatal mortality, birth weight, breast and infant feeding, and immunization coverage were excluded; while the evidence remains limited on the scale and nature of the impact of armed conflict on these indicators, child nutrition and maternal and newborn health are broadly recognised as carrying high risk in conflict settings.[23, 24] However, if these data were presented together with other child health and development outcomes, then data for all reported child health and development outcomes were extracted. Studies on the effects of exposure to the atomic bomb were excluded, as there are existing reviews on this subject. Post war studies were included if they provided associations of the outcomes with armed conflict. No restrictions were made for sex, geographic location, language, or study design.

The risk of bias was assessed at the study ad outcome levels for each individual study based on the data source, study population, sampling strategy, data collection and analysis methods, and any special characteristics of the population. Studies that were deemed to have unsound or invalid methods were excluded. Given the challenges in obtaining data in conflict settings, studies from single facilities, studies using only facility-based data, and case reports were included. Data from studies meeting inclusion criteria were abstracted onto a data extraction form, including time period, study country and sub-region, identified conflict, study design, reference population, type of exposure, health outcomes, access to basic needs, mortality, and associations between exposures and outcomes. Where available, data were abstracted for protective and mitigating factors on child health outcomes. When possible, authors were contacted for missing data. In the case of queries or differences, an agreement was negotiated between the reviewers.

### Role of the funding source

The University of Florida-Jacksonville provided partial funding for the study. There was no additional external funding received for this study. The study design, data collection, analysis, interpretation of data, and writing of the report were undertaken independently. All authors had full access to all the data in the study and they shared responsibility for the decision to submit for publication.

## Results

**Fig 2:**
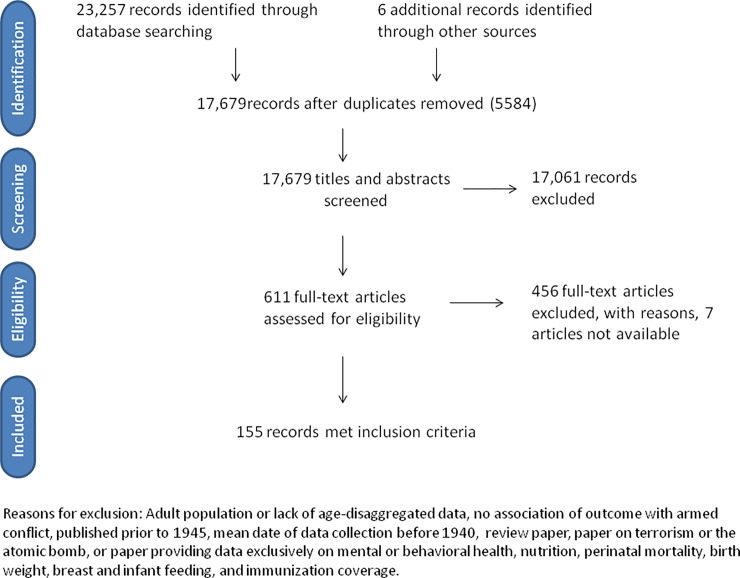
Flow diagram.

The searches retrieved 23,257 records, and six additional papers were retrieved through snowball and hand searching. After removal of duplicates, 17,679 titles and abstracts were screened. Of 618 papers eligible for full-text review, 7 were not available. 611 papers were reviewed in full text. 456 studies were excluded, with reasons (Fig 2). Our final sample consisted of 155 studies, including 18 published between July 2015 and April 2017.

**Fig 3:**
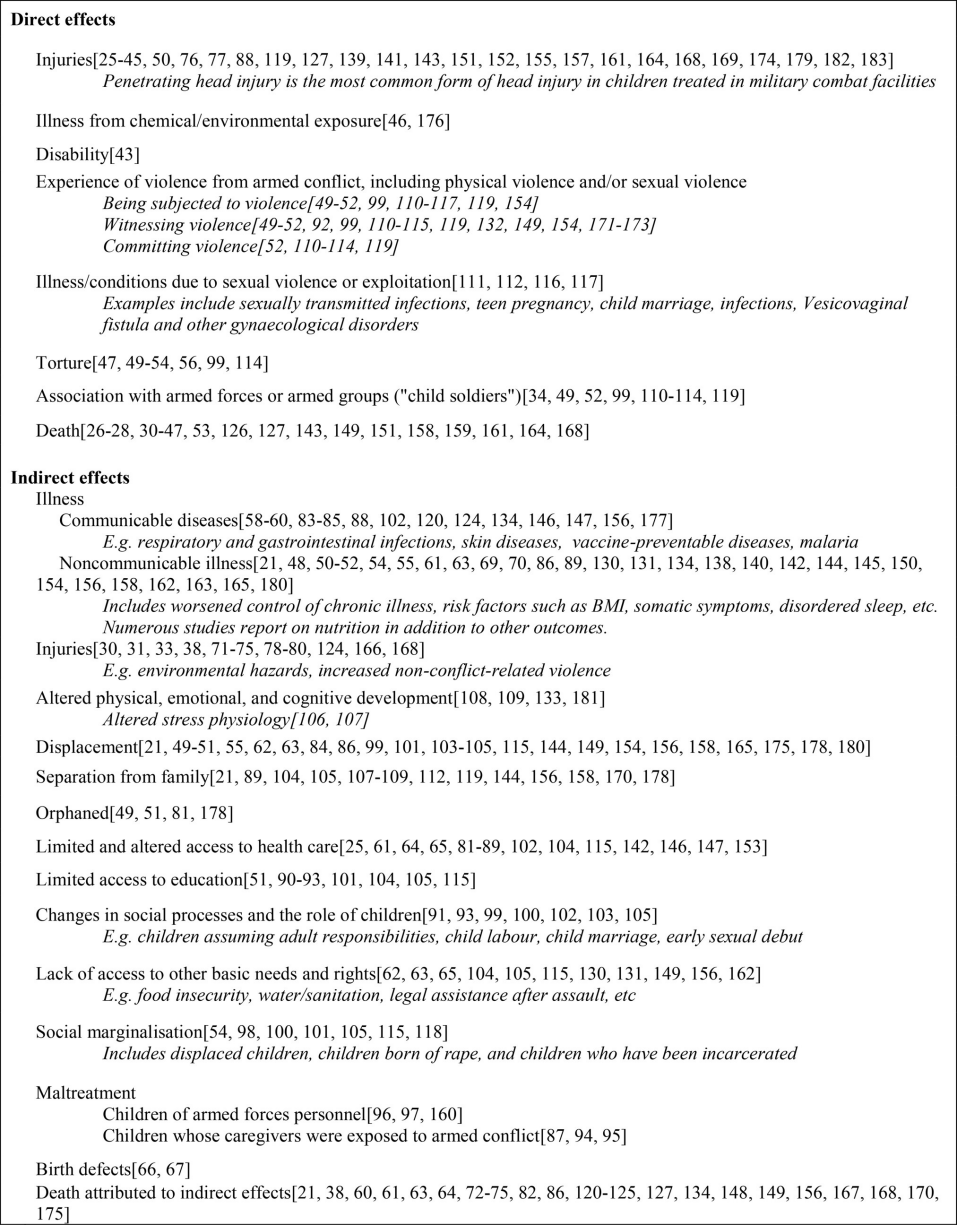
Summary of principal findings reported in the literature.

Among the included publications, 131 were quantitative studies, 18 qualitative, and six were mixed methods design. Included in the quantitative studies were 20 case reports, 44 chart or registry reviews, and 48 cross-sectional studies. The data from these studies is too heterogeneous to be pooled for meta-analysis. Collectively, they provide a body map of the child affected by armed conflict (Table 1 and Fig 3).

**Table 1:** Summary of included studies.

**Studies published 1 January 1945–12 June 2015**					
	**Autdor**	**Conflict zone**	**Population**	**Sample size**	**Summary of findings**
1	Aaby et al,[21] 1999	Guinea Bissau	IDP and resident children	422	During the period of displacement, child mortality increased and nutritional status deteriorated for both IDP children from Bissau and resident children. Mortality for resident children was 4.5 times higher, decline in growth was significantly worse, and recovery later than for displaced children.
2	Abushaban et al,[66] 2004	Kuwait	Kuwaiti infants with CHD	2,256	2,256 babies with congenital heart disease (CDH). The mean annual incidence of CHD rose from 39.5 per 10 000 live births pre-war (1986–1989) to 103.4 per 10 000 live births post war (1992–2000)
3	Amitai et al,[138] 1992	Israel-Palestine	Emergency department cases	268	Accidental atropine poisoning in children from atropine autoinjector provided to families in case of organophosphate nerve gas attacks.
4	Araneta et al,[67] 2003	Iraq	Infants born to US military personnel who served in February 1991	45,013	Increased prevalence of congenital disorders in infants conceived post-war to Gulf War veterans (GWV). Infants of GWV men had increased rate of tricuspid insufficiency or regurgitation, aortic stenosis, and renal agenesis or hypoplasia. Infants of female GWVs had higher prevalence of hypospadias.
5	Ascherio et al,[60] 1992	Iraq	National	16,076	Three-fold increase in infant and child mortality after start of conflict. Four-fold and five-fold increases in age-adjusted mortality from injuries and diarrhoea, respectively. Regional differences in child mortality were maintained or exacerbated after onset of conflict.
6	Avogo and Agadjanian,[86] 2010	Angola	Migrants to Luanda	719	Increased mortality among children whose families were displaced due to war. This effect was strongest during the first year after migration.
7	Barisić et al,[139] 1999	Former Yugoslavia	Children with nerve injuries	27	Peripheral nerve injuries in children due to war involved multiple nerves, were located proximally on upper extremities, had complete or severe nerve damage, delayed reinnervation and poor spontaneous recovery outcomes. These patterns differed from children with peripheral nerve injury due to accidents, who primarily had single, partial peripheral nerve injuries that were located on distal extremities.
8	Barnes et al,[140] 2007	Iraq	US high school students	121	Children of deployed military personnel had significantly higher BMI than non-deployed and civilian counterparts. Children of both deployed- and non-deployed military personnel had a higher mean HR than children of civilians.
9	Bertani et al,[141] 2015	Afghanistan	Patients <16 years in a NATO military combat hospital	89	Injuries due to explosive device were more common in children than from firearms and were associated with a high rate of both traumatic and surgical amputation. All fractures were open fractures, with high rates of vessel and nerve injuries.
10	Betancourt et al,[111] 2008	Sierra Leone	Former child soldiers	260	Child soldiers describe witnessing violence, becoming soldiers for survival, child labour, sexual violence, and unwanted pregnancy.
11	Betancourt et al,[110] 2010	Sierra Leone	Former child soldiers	156	Average abduction age 10.5 years, nearly all were forced into conscription, nearly all reported witnessing violence, more than 1/4 had killed someone (including a loved one) and 1/3 of girls reported being raped
12	Bilukha and Brennan,[71] 2005	Afghanistan	Injuries due to landmine or UXO	6,114	UN database, 1997–2002. 54% of UXO injuries were in children, the majority between ages 5–14 years. Of these, 42% were injured while playing with the device”
13	Bilukha et al,[73] 2003	Afghanistan	Injuries due to landmine or UXO	1,636	ICRC database 2001–2002: 46% of UXO injuries were in children under 16 years. 49% of children injured were playing or tending animals.
14	Bilukha et al,[75] 2006	Chechnya	Civilian injuries due to landmine or UXO	3,021	Region wide data, 1994–2005. 26% of UXO injuries were in children under 18 years, with 17% mortality rate in these children. 35% of children with upper body injuries, 20% with lower body injury, 24% with both upper and lower body injury, and 26% had limb amputations.
15	Bilukha et al,[72] 2008	Afghanistan	Injuries due to landmine or UXO	5,471	ICRC database, 2002–2006. 47% of injuries and deaths were in children. 42% of child UXO injuries were upper body and 27% were both upper and lower body injuries. 2/3 of child injuries occurred during active hostilities. 2/3 were in children tending animals or tampering or playing with an explosive device
16	Bilukha et al,[74] 2011	Nepal	Injuries due to landmine or UXO	307	National prospective surveillance 2006–2010. 55% of injuries were in children under 18 years, with 15% mortality rate in these children. Nearly two-thirds of child injuries occurred while playing or tampering with an explosive device, the greatest number in children aged 10–14 years old. 40% of explosions occurred in victim's homes.
17	Bodalal et al,[142] 2014	Libya	Deliveries at a single health facility	13,031	Prevalence of preterm deliveries and LBW increased during the conflict when compared with pre-war. There was a higher rate of caesarean section delivery and episiotomy during the conflict.
18	Bogdanovich and Schevchenko,[25] 1946	WWII	Paediatric eye injuries at a single tertiary facility	220	Incidence of ocular trauma increased 3.2 during armed conflict compared with peacetime. 84% of cases were in school-aged children.85% injuries were from weapons of war. Half of children presented 6 days or later after injury.
19	Borgman et al,[41] 2012	Afghanistan and Iraq	Paediatric patients treated at US military combat facilities	7,505	Data from Patient Administration Systems and Biostatistics Activity Database (PASBA) and Joint Theatre Trauma Registry (JTTR). 79% of paediatric admissions were due to trauma. Paediatric trauma patients had higher mortality and longer hospital stays than adult comparison groups. Most common Injury mechanisms were blast, penetrating, blunt trauma, and burns. Children under <8 years had higher mortality than children >8 years.
20	Borgman et al,[143] 2015	Afghanistan and Iraq	Isolated burn patients treated in US military combat facilities	4,743	Paediatric burns patients in conflict zones have higher mortality compared with patients in the United States.
21	Bosnjak et al,[89] 2002	Former Yugoslavia	Children with seizures in two facilities	111	Displaced children from war-affected areas had worsened epilepsy control, with greater frequency of epileptic seizures and less regular follow up. These children were also more likely to be separated from their fathers or both parents than children from areas not directly affected by the war. Medication compliance was similar in both groups.
22	Bronstein and Montgomery,[144] 2013	Afghanistan	Unaccompanied minors in state care in London	222	Nearly 2/3 of children reported sleep disturbance in the form of nightmares
23	Busby et al,[145] 2010	Iraq	Households in Fallujah	711	Risk ratio 12.6 for childhood cancer in children in Fallujah 0–14 years compared with peers in Egypt and Jordan.
24	Celikel et al,[26] 2015	Syria	Syrian children injured due to war in Syria who died in a single Turkish facility	140	18% of in-hospital deaths were children, median age was 12. 70% of injuries were from bombing and shrapnel, 13.6% gunshot wounds, 13.6% blunt force and 2.8% motor vehicle crashes while trying to escape. 2/3 of paediatric deaths were due to head injuries, and 30% had isolated head and neck injuries
25	Chironna et al,[146] 2001	Kosovo	Kosovar refugee children and youth in camps in Italy	371	251 children ≤10 years, 119 were 11–20 years. Hyperendemic Hepatitis with exposure in early childhood. 61% of children aged 2–1 0 years were seropositive and 100% of children over 11 years were HAV seropositive. 11% of children 2–10 years were HBV positive and 39% of children and youth aged 11–20 were HBV positive. No children had been vaccinated against HBV.
26	Chironna et al,[147] 2003	Iraq and Turkey	Kurdish refugee children and youth in camps in Italy	269	98 children ≤10 years, 171 were 11–20 years. Hyperendemic Hepatitis A, High seroprevalence of Hepatitis E, with 89% of Hepatitis E exposed from Iraq. Hepatitis B was also endemic, and children from both Turkey and Iraq had low vaccination rates against Hepatitis B.
27	Cliff et al,[130] 1997	Mozambique	Children in Mogincual district, Mozambique	228	High rates of malnutrition in war-affected areas, with a two-year outbreak of clinical Konzo. Konzo was attributed to shortened cassava processing due to war-related disruption
28	Cohn et al,[48] 1979	Chile	Chilean refugee children in Denmark	75	All children were either tortured, had a parent who was tortured, or had a parent who was imprisoned in Chile. One third of children had sleep disturbances including nightmares and difficulty falling asleep. 1/4 reported nocturnal enuresis. Numerous somatic complaints, including anorexia, headaches, abdominal pain, difficulty concentrating, impaired memory, and constipation.
29	Coppola et al,[27] 2006	Iraq	Paediatric cases treated at a US military combat facility	85	Data from surgical logs. Patterns of trauma included fragmentation wound (52%), penetrating trauma (23%), burn (19%), and blunt trauma (6%). The primary injury site was the lower extremities in 38%, followed by head injury (23%).
30	Cowan et al,[68] 1997	Iraq	Live births to military service members	75,461	No elevated risk of birth defects, reduced fertility or differences in sex ratio was found among veterans of the first Gulf War.
31	Creamer et al,[28] 2009	Afghanistan and Iraq	Paediatric patients treated in US military combat facilities	2,060	Data from PASBA database. Children accounted for a tenth of all combat support hospital admissions, the majority of which suffered penetrating trauma. Gunshot wounds were more common in Iraq, while burns and landmine injuries were more common in Afghanistan. Younger age was associated with higher mortality.
32	Curlin et al,[120] 1976	Bangladesh	Population in Matlab Bazaar, Bangladesh	120,000	During the war, overall infant mortality rate rose 15% above baseline, of which the post-neonatal infant mortality rose 46%. Mortality in 1–4 year olds rose 43%. Mortality for all U5 subgroups returned to baseline during the year after the war. Among children 5–9 years olds, mortality rose 208% during the war and continued to rise during the year after, to 281% above baseline, attributed to smallpox and dysentery epidemics.
33	de Smedt,[103] 1998	Rwanda	Rwandan refugees in Tanzania	6	Describes complex changes in social norms after the displacement due to conflict. Child marriage became common, with the median age of girls 15 years, and 15 years for boys. Marriages were reported in girls as young as 13 and boys as young as 14.
34	Deeb et al,[148] 1997	Lebanon	Children in Beirut	4101	Muslim children had 1.6 times the risk of dying before 5 years compared to Christians. When controlling for the number of children ever born to the mothers, elevated risk of U5 mortality remained significant for lowest social class of Muslims only.
35	Denov,[112] 2010	Sierra Leone	Former child soldiers	80	Describes children’s involvement with armed groups in detail. Children acted as front-line combatants, commanders of other child soldiers, spies, porters, cooks, domestic servants, and care-takers of younger children. The children described being subjected to extreme physical, psychological, and sexual violence, as well as injuries, chronic pain, loss of family and social and economic marginalisation.
36	Depoortere et al,[149] 2004	Darfur, Sudan	IDPs in Darfur	17,339	In two villages affected by armed conflict, 5% and 34.5% of violent deaths were in children under 15. One refugee camp had markedly elevated non-violent mortality, of which 48% of deaths were in children under 5 years
37	Devanarayana and Rajindrajith,[150] 2010	Sri Lanka	Children in two schools in war affected regions	2,699	Living in a war-affected area was independently associated with constipation (adjusted OR 1.48).
38	Di Maio and Nandi,[91] 2013	Israel-Palestine	Palestinian boys 10–14 years in the West Bank	45,419	Closure of the border between Israel and the West Bank significantly increased the probability of child labour. A 10 day increase in the quarterly number of school closure days increased the probability of child labour by 11%.
39	Dickson-Gómez,[99] 2002	El Salvador	Former child soldiers	4	Describes the experiences of child soldiers including witnessing and being subjected to torture, imprisonment, displacement, loss of family, being orphaned, assumption of adult roles and responsibilities, child marriage, teen pregnancy, and difficulty reintegrating into their communities.
40	Edwards et al,[151] 2012	Afghanistan	Trauma patients in US military combat facilities	1,205	Data from JTTR. Children <15 years accounted for more than half of severe head and neck injuries. Children ≤7 years or younger were more likely injured by mortar fragments, while children 8–14 years were higher risk for land mine or UXO injury.
41	Edwards et al,[152] 2014	Afghanistan and Iraq	Trauma patients in US military combat facilities	1,205	Data from JTTR. Children <15 years accounted for 25% of civilian admissions 2002–2010. Infants and young children ≤3 years most often required neurosurgical procedures. Extremity amputation and external fixation were more common in children >4 years
42	Eide et al,[153] 2010	multiple	Children of US military personnel	169,986	During deployment, children of single parents had lower outpatient visit rates while children of married parents had increased visit rates. Parent age <24 years and single marital status were associated with lower visit rates. There was an overall increase in outpatient visits and well-child visits during deployment compared with periods when the parent was not deployed.
43	Elbert et al,[154] 2009	Sri Lanka	5th graders in north-eastern Sri Lanka	420	77% of children had been displaced at least once. 92% had experienced violence from the war including combat, bombing, shelling, or witnessing the death of a loved one.
44	Erjavec and Volcic,[100] 2010	Former Yugoslavia	Adolescent girls born of war-related rape	11	The adolescents describe social isolation from the mother's ethnic group, as well as being assaulted, shot at and threatened with rape. They also described taking on the role of carer for incapacitated mothers.
45	Feldman et al,[106] 2013	Israel-Palestine	War-exposed and non-exposed children 1.5–5 years	232	War-exposed cohort had lower baseline cortisol levels and less reactivity to stress than the non-exposed cohort. Children’s baseline cortisol levels were independently related to maternal baseline cortisol lower maternal reciprocity, and greater maternal psychopathology.
46	Garfield and Leu,[121] 2000	Iraq	Children U5	8,191	Used MICS 1996 survey data. U5 mortality more than tripled during the war, then fell afterwards during post-war conflict and sanctions, but remained at least twice the pre-war level.
47	Gasparovic et al,[76] 2004	Former Yugoslavia	Case report	1	9 year old girl with injuries due to UXO, including intracranial shrapnel, intracardiac shrapnel, multiple intestinal perforations, multifragmentary fracture of the right distal humerus, and explosive injuries of the soft tissues of the right thigh and right foot.
48	Gataa and Muassa,[155] 2011	Iraq	Patients at 2 facilities	551	One-fifth of patients with maxillofacial injuries were children <15 years
49	Geltman et al,[50] 2005	Sudan	Sudanese unaccompanied minors in foster care in the US	304	One fifth of the youth reported being tortured and 29% sustained war-related physical injuries. They reported near-drowning, near-suffocation, head trauma, and loss of consciousness. The youths reported seeking health care for a variety of somatic symptoms including headaches, stomach aches, anorexia, and chest pains. Nearly 1/3 had sleep disturbances.
50	Gessner,[62] 1994	Afghanistan	Displaced and resident families in Kabul	612	Displaced families lived with a mean of 15 people per room compared with residents, who had a mean of 2 people per room. The average number of people per toilet for displaced persons was 44, and two locations for displaced people had no working toilets.
51	Ghobarah et al,[57] 2004	multiple	Populations in countries after civil war	Not specified	Significant reduction in DALYs for all disease categories in children <15 years, most commonly due to infectious diseases, and with the most severe reductions in children under 5. This effect was found for countries experiencing civil war as well as countries adjacent to them.
52	Goma Epidemiology Group,[156] 1995	Rwanda	Children in three refugee camps	Not specified	Mortality estimates ranged 20-120/10,000/day for unaccompanied children and 100-800/10,000/day unaccompanied infants. High rate of death attributed to diarrhoea. .
53	Green,[51] 2007	multiple	Case report	3	3 child victims of torture, reported child labour, slow insertion of a knife into the child's thigh to extract information from the parents, and witnessing torture, including witnessing a parent tortured to death. The children reported recurrent nightmares and school absenteeism.
54	Greene et al,[157] 2014	Iraq	Case report	1	3 year old girl with blast injuries to the right arm and chest, who required highly specialised thoracic surgery and was hospitalised for 16 days.
55	Grein et al,[158] 2003	Angola	Refugees in 4 camps	6,599	18% of the population was U5. U5 mortality was four times above baseline. Main causes of child death were malnutrition, fever and malaria. Children accounted for one-fourth of deaths related to war violence, and 55% of disappearances.
56	Guha-Sapir and van Panhuis,[123] 2003	multiple	Children in multiple conflict zones	Not specified	Analysed data from mortality surveys in 7 populations in Afghanistan, the Democratic Republic of Congo, Somalia, and Sudan. The relative risk of dying during conflict differed based on context and appeared to be associated with the fragility of the affected population.
57	Guha-Sapir and van Panhuis,[122] 2004	multiple	Populations affected by armed conflict	Not specified	In pooled data from 37 datasets, children ≥5 years have a higher relative risk of dying during conflict compared to children U5. There were wide variations in mortality rates between conflicts.
58	Guy,[52] 2009	DRC	Case report	3	The 3 children were subjected to physical and psychological torture and reported participation in violence. The three children had a combined total of 403 medical complaints. Clinical exam revealed 275 physical findings that were consistent with the torture mechanisms described by the children.
59	Hagopian et al,[69] 2010	Iraq	Child cancer patients at a single facility	698	Leukaemia incidence in children aged <15 years presenting to the referral facility in Basra more than doubled over the period 1993–2007.
60	Halileh and Gordon,[131] 2006	Israel-Palestine	Children 6–59 months in Gaza and the West Bank	3,331	37% of children were anaemic, with linear decrease in prevalence of anaemia with increasing age. Independent risk factors included age <24 months, living in refugee camps, living in Gaza, and household income being affected by the conflict.
61	Hanevik and Kvåle,[77] 2000	Eritrea	Landmine injury patients in 5 hospitals	248	Chart review of 248 patients with landmine injuries. 41% of patients were under 15 years, and 22% were 15–19 years old.
62	Helweg-Larsen et al,[29] 2004	Israel-Palestine	Patients presenting to two emergency departments	962	Chart review of intentional injuries pre-conflict and during Intifada. Marked rise in intentional injuries during conflict. 3% of patients were <10 years old and 8% of patients were 10–14 years old. Head injuries were mostly caused by firearms and were more frequent among the very young children.
63	Henrich and Shahar,[132] 2013	Israel-Palestine	7th-10th graders in southern Israel	362	Greater rocket attack exposure predicted an increased aggression in boys, with increased odds of committing violence over the course of the study.
64	Hicks et al,[53] 2011	Iraq	Iraqi civilians killed during the conflict	92,614	Data from Iraq Body Count database 2003–2008: 2,146 (3.5%) deaths were children under age 18. 36,900 (61.0%) civilian victims of unreported age. Children were killed by all measured methods, including execution, execution with torture, small arms gunfire, suicide bomb, vehicle bomb, roadside bomb, mortar fire, and air attacks both with and without ground fire
65	Hicks et al,[159] 2011	Iraq	Death or injury due to suicide bomb	42,928	Data from Iraq Body Count database 2003–2010: Children accounted for 14% of deaths due to suicide bombs and for a higher proportion of deaths due to suicide bombs than from general armed violence. 16% of suicide bomb events resulted in the death of at least one child.
66	Hisle-Gorman et al,[160] 2015	multiple	Children 3–8 years old of deployed US military personnel	487,460	Parental deployment was associated with increased rates of child maltreatment. For the children of injured veterans, there was a 24% increase in child maltreatment visit rates for each additional parent injury diagnosis.
67	Hoffer and Johnson,[161] 1992	Iraq	Children with shrapnel wounds at US military combat facility	19	19 of >100 children with shrapnel wounds had associated open fractures. Mechanism of injury included UXO, aerial bombardment, and bullet wounds from combat. Open fractures from shrapnel were most common in the tibia and fibula, and 9 children had bilateral fractures from shrapnel.
68	Inwald et al,[42] 2014	Afghanistan	Intensive care patients at a British military combat facility	811	Data from Bastion intensive care unit database and JTTR. 14% of intensive care admissions were children, median age 8 years. 71% were trauma admissions, of which 65% had blast injuries, 20% gunshot wounds, and 15% blunt trauma. Body region of trauma: 45% extremities, 35% face/eyes, 26% head, 20% abdomen, 11% thorax, and 4% pelvis
69	Kandala et al,[59] 2009	DRC	Children U5 in DRC	9454	Higher prevalence of childhood diarrhoea, acute respiratory infection and fever in provinces experiencing armed conflict
70	Kinra and Black,[78] 2003	Former Yugoslavia	Landmine injury patients in Bosnia	4,064	ICRC database 1991–2000 (during and post-conflict). 14% of patients were children, the majority who were injured during recreational activities. Children were more likely to be injured during peacetime when compared with adults.
71	Klimo et al,[31] 2010	Afghanistan	Neurosurgical patients at a US military combat facility	43	Data from surgeon’s personal records. 40% of paediatric patients (<18 years) were under 5 years of age The average age of paediatric patients was 7.5 years. Penetrating brain injuries were most common. IED most frequent source of projectile, followed by rocket, landmine, mortar and gunshot wound.
72	Kvaskoff et al,[162] 2013	WWII	Cohort of French women born in 1925–1950	75,918	There was a linear relationship between the level of World War II food deprivation before 20 years of age and endometriosis risk.
73	Lee et al,[124] 2006	Myanmar	Households living in conflict zone	1290	Infant mortality rate in the conflict zone was nearly double and U5 mortality rate was nearly triple the level of the national rates.
74	Liu et al,[163] 2014	multiple	Live births to war refugee women in Sweden	20,723	Significantly higher odds of preterm (OR 1.39, 95CI: 1.13–1.72) or very preterm birth (OR 2.15, 95CI: 1.37–3.38) during the 1st year of residency in Sweden. The risk continued beyond the first year, with increased risk of very preterm birth (OR = 1.54 95% CI 1.07–2.21) during the third to fifth year of residence.
75	Longombe et al,[116] 2008	DRC	Child rape survivors	7	Case report of the gang rapes by armed forces of a 6 year old and a 12 year old, respectively. The children developed vesicovaginal fistulae.
76	Maclure and Denov,[113] 2006	Sierra Leone	Former child soldiers	36	Description of the children's experiences, including physical abuse, psychological torture, witnessing of violence, drug use, child labour, and the use of children as human shields.
77	Mann,[101] 2010	DRC	Congolese undocumented refugee children in Tanzania	>100	Children describe social isolation, stigmatization, dehumanization, harassment and xenophobia, child labour, exploitation, and physical abuse. Fear of being reported to the police, imprisoned or deported. The children also describe lack of agency and barriers in access to education.
78	Manoncourt et al,[63] 1992	Somalia	IDPs and residents in Mogadishu	4169	High child mortality rates (per 1000 live births), which varied by IDP status and residence. IDPs in camps with U5 mortality rate (MR) 240.6 (95CI: 206.0–275.2), and 5–14 MR 167.1 (95CI: 140.5–193.7). IDPs in towns with U5MR 86.2 (95CI: 50.1–122.3), and in 5–14 MR 89.7 (95CI 52.2–127.2). Residents with U5MR: 115.4 (95CI: 85.6–145.2), 5–14 MR 57.8 (95CI: 38.7–76.9). Markedly limited access to water for IDPs in camps.
79	Martin et al,[32] 2010	Iraq	Paediatric neurosurgical patients at a US military combat facility	42	Prospective study. 15% of civilian patients were children <18 years, 62% of whom were ≤8 years. 52% had penetrating head injuries, 24% had closed head injuries, and 12% had penetrating spinal injuries. 22% overall mortality in children, and 32% mortality in children with penetrating head injuries.
80	Masterson et al,[87] 2014	Syria	Syrian refugee women in Lebanon	452	Describes limited access to antenatal and delivery care, with high rates of complications and adverse birth outcomes, including low birth weight, preterm delivery, and infant mortality. Less than half reported any breastfeeding, citing inability to breastfeed, illness, and constant displacement as reasons. 75.8% reported beating their children more than usual.
81	Mathieu et al,[33] 2015	Afghanistan	Combat facility paediatric patients with extremity trauma	155	Mean age of patients was 9 years. 46% were <8 years. Younger children more likely to have noncombat-related injuries (NCRI). Male to female ratio 3:1. 77 combat-related injuries (CRI) including 19% bullet wounds, 42% fragment wounds, 39% blast wounds. Motor vehicle crashes, falls and burns were the most common noncombat-related mechanism of injury. Injury severity score significantly higher for CRI.
82	Matos et al,[34] 2008	Iraq	Paediatric trauma patients treated in a US combat facility	1132	Data from hospital records. 97% of patients were >8 years old. 63% of young children (≤8 years) and 83% of older children and adults had penetrating injuries. Young children had more severe injuries. Young age was independently associated with higher mortality.
83	McGuigan et al,[35] 2007	Iraq	Paediatric trauma patients treated in a US combat facility	99	Data from hospital records. 55% of patients were <13 years old. 79% of injuries were due to battle or crossfire, the majority were blast wounds or penetrating trauma. None of the children had protective armour, and most had multiple injuries. Blast victims had a combination of blunt, penetrating, and thermal injuries. 9% mortality.
84	Metreveli and Vosk,[164] 1994	Republic of Georgia	Child casualties in Georgian Civil War	5	Case report of five children injured or killed by hand grenades or firearms during the civil war. Treatment was hampered by a shortage of medical equipment and supplies.
85	Miller et al,[88] 1996	Former Yugoslavia	Children treated in a referral facility, Sarajevo	60	Marked reduction in services, number of beds, number of staff, access to medications, barriers to care and length barriers to returning to family, for up to 18 months, due to the war. Of hospitalised children, 20% had experienced the death of a close relative in the war and 38% had one or more close family members injured.
86	Momeni and Aminjavaheri,[46] 1994	Iran-Iraq	Children exposed to mustard gas	14	Compared with adults, the children had earlier onset of symptoms and a different clinical pattern. Children's first symptoms were cough and vomiting. Facial involvement was most common. Skin bullae appeared sooner, and the children developed more severe ophthalmic manifestations.
87	Montgomery and Foldspang,[165] 2001	multiple	Child asylum seekers in Denmark	311	Predictors of sleep disturbance (frequent nightmares, delayed sleep onset, and night-time awakenings) included violent death of grandparents before the child's birth, torture of one or both parents, and being scolded more than previously. Being accompanied to Denmark by both parents reduced the risk of sleep disturbance (OR 0.3 and p<0.0005).
88	Mujkic et al,[166] 2008	Former Yugoslavia	Child deaths due to injury 1986–2005	4,660	During the war, the rates of child homicide and suicide using weapons more than tripled and unintentional child deaths with weapons increased more than 6-fold compared to pre-war period. After the war, these rates gradually returned to pre-war levels.
89	Nelson et al,[117] 2011	DRC	Child survivors of sexual violence	389	Children <18 years were more likely than adults to have been gang raped, raped by a civilian, and raped during the day. Education was protective. The study found an increase in civilian-perpetrated rape during conflict. Nearly 1/4 of child rape survivors had physical sequelae and 19% reported pregnancy resulting from rape. 18% of the children reported efforts to bring the perpetrator to justice, most often with civilian perpetrators.
90	Nicaragua Health Study Initiative,[81] 1989	Nicaragua	Households in two towns	89	Comparison of a town in a peaceful region with a town in a conflict zone. Children in town experiencing armed conflict had higher prevalence of stunting, lower odds of having an immunization card, lower rate of U5 vaccination completion, and twice the odds of being an orphan.
91	Nielsen et al,[126] 2006	Guinea-Bissau	Children <5 years near Bissau	8933	U5 mortality doubled during the first six months of the war. In the later part of the war, U5 mortality began to return to baseline, but mortality for girls remained significantly higher than pre-war. Maternal education was protective against U5 mortality.
92	Novo et al,[85] 2009	Former Yugoslavia	Rubella cases in Bosnia	342	Post-war rubella outbreak investigation revealed that MMR vaccination coverage dropped from 93.6% in Bosnia pre-war to 56.8% during the last two years of the war. Partially- and unvaccinated patients never received catch-up vaccinations.
93	O'Hare and Southall,[82] 2007	multiple	Children in Sub-Saharan Africa	Not specified	Data from UNICEF SOWC 2006 report in 42 Sub-Saharan African countries. Median U5 mortality rate in countries with recent conflict was 197/1000 live births, significantly higher than countries without recent conflict (137/1000 live births, p = 0.009). Women in countries with recent conflict were less likely to deliver with a skilled birth attendant, 1 year olds had lower DTP vaccination rates, and primary school enrolment was lower.
94	Pannell et al,[36] 2015	Afghanistan	Paediatric trauma patients in a NATO combat facility	263	Data from JTTR. 11.7% of trauma patients were children, median age 9 years (range 3 months—17 years), nearly 1/3 were under 6 years. 62% had battle injuries. Injury mechanism: 42% blast injuries, 17% GSW, 16% motor vehicle crash, 8% falls and 4% burns. More than half of children had penetrating injuries. ISS was higher for children <15 years. 8% inpatient mortality.
95	Patel et al,[104] 2012	Uganda	IDPs in northern Uganda	>116	116 In-depth interviews and 16 focus group discussions. Displacement and low security led to disruption of community structure and social norms; this was associated with changes in sexual behaviour. Girls living in IDP camps were vulnerable to sexual exploitation, violence, economic and food insecurity, and had barriers in access to education and health care.
96	Pesonen et al,[108] 2008	WWII	Helsinki birth cohort	1,704	Girls separated from their parents in early childhood were twice as more likely (OR 2.1, 95CI: 1.2–3.7) to have their menarche before or at the age of 12 than after the age of 13, compared with non-separated girls.
97	Pesonen et al,[107] 2010	WWII	Helsinki birth cohort	282	Adults who had been separated from both parents during early childhood had higher salivary cortisol and plasma ACTH concentrations and greater salivary cortisol reactivity to during Trier Social Stress Test compared with non-separated. Gendered differences were found in baseline cortisol, ACTH, and cortisol reactivity. Age at separation from both parents predicted salivary cortisol, plasma cortisol, and plasma ACTH.
98	Pesonen et al,[109] 2011	WWII	Helsinki birth cohort	2,725	Young men separated temporarily from both parents during early childhood had lower intelligence scores than non-separated men. Findings differed by length of separation and age at the time of separation. Verbal ability was particularly impacted in boys separated from their parents before school age.
99	Poirier,[90] 2012	multiple	Children in Sub-Saharan Africa	Not specified	Data from 1950–2010 in 43 countries. Armed conflict and military expenditure increase the rate of children not attending school and have a negative effect on secondary school enrolment rate.
100	Qouta et al,[133] 2008	Israel-Palestine	School children in Gaza	865	Witnessing severe military violence was associated with aggressive and antisocial behaviour in school children. Parenting practices appear to be a moderating factor.
101	Radoncic et al,[167] 2008	Former Yugoslavia	Births at a tertiary facility in Bosnia	101,712	Perinatal mortality was decreasing before the war. There was a significant increase during the war and early post-war period and decline again to pre-war levels in 2001. The main causes of perinatal mortality were respiratory distress syndrome, birth asphyxia, congenital malformations, and intracranial haemorrhage.
102	Radonic et al,[79] 2004	Former Yugoslavia	Patients with antipersonnel mine injuries	422	Data during the war 1991–1995 in southern Croatia. Children accounted for 7.8% of antipersonnel mine injuries and were most commonly injured while playing with the mines, on the way to school, or near their home. The mean age of injured children was 10.5 years.
103	Rashid,[54] 2012	Kashmir, India/ Pakistan	Children who were detained and tortured in Kashmir	43	Data from personal accounts of children-in-conflict-with-law in Indian-held Kashmir. The children describe lengthy imprisonments in crowded facilities together with adults, forced labour, and inadequate food. Torture methods described included being stripped, blindfolded, having limbs stretched, electrocution of private parts and of limbs, hanging from ceiling by arms, beatings, rollers, breaking teeth, and threats to family. The children describe poor physical health and social isolation after release.
104	Rees et al,[94] 2013	Timor-Leste	Women in an urban area and a rural village	1513	Victimization and war-related trauma increased the odds of Intermittent explosive disorder in women. Women with IED reported excessive and harmful punishment of their children.
105	Rentz et al,[97] 2007	multiple	Children <18 years who experienced substantiated maltreatment in 2000–2003	147,982	Texas child maltreatment data from 2000 to 2003: Substantiated child maltreatment in military families doubled after October 2002 (Rate ratio 2.15, 95CI: 1.85, 2.50), after controlling for child’s age, race/ethnicity, and gender. The rate of child maltreatment in military families increased by approximately 30% for each 1% increase in the percentage of active duty personnel departing to or returning from operation-related deployment. The majority of child maltreatment in military families was perpetrated by a parent.
106	Reyna,[168] 1993	Iraq	Paediatric patients treated in a US combat facility	50	Data from hospital records. Description of 50 paediatric patients 0–19 years seen at an evacuation hospital in Kuwait. 80% were trauma patients, of which 65% had penetrating injuries. Injuries included shrapnel wounds, gunshot wounds, burns, motor vehicle accidents, crush injuries, and falls. There were no trauma-related deaths.
107	Roberts et al,[127] 2004	Iraq	Households in Iraq	6300	Household survey found that crude infant mortality increased from 29 per 1000 live births pre-invasion to 57 deaths per 1000 live births post-invasion [95% CI 0–64]. Violence accounted for more than half of recorded child deaths in the post-invasion.
108	Rodríguez and Sánchez,[93] 2012	Colombia	Children 6–17 years in Colombia	20,642	National survey found that violent attacks significantly increase the risk of school drop-out. An increase by one standard deviation of armed conflict exposure increased the joint probability of child labour and school drop-out by 13% in children 12–17 years. The effect appeared to be long-term, with adults in areas affected by conflict having lower educational outcomes.
109	Saile et al,[95] 2014	Uganda	Households in Gulu and Nwoya districts	283	Traumatic war exposure in female guardian independently predicted child-reported experiences of abuse in the family. Partner violence between guardians and PTSD-symptoms in male guardians were the major proximal risk factors for child-reported victimization, suggesting that war exposure and subsequent trauma may be a mediating factor in violence against children.
110	Salignon and Legros,[134] 2002	Republic of Congo	Residents of Mindouli	10,026	83.5% of the population were displaced by the war and had returned to their homes in Mindouli during the 3 months preceding the survey. 195 U5 deaths were reported in the 6 months preceding the survey, accounting for 13% of the U5 population of Mindouli in November 1999. The U5 mortality rate exceeded 10 deaths/10,000/day November 1999-January 2000. Causes of U5 death were malnutrition (54,4%), fever (17.4%), diarrhoea (6.7%), and 21.5% other cause
111	Samms et al,[169] 2010	Iraq	Case report	1	16 year old with a gunshot wound to the pelvis and possible blast injury to the abdomen. The patient had an 83 day hospital course, required 30 operations, and was discharged to a local hospital.
112	Santavirta,[170] 2014	WWII	People born in Finland 1933–1944 registered in the 1950 census	66,053	Men who were evacuated to foster care in Sweden at age <4 years had mortality risk 1.3 times higher than their counterparts who were not evacuated. There were no other significant mortality differences based on gender, age at time of evacuation, or between evacuation-status discordant siblings.
113	Schiff et al,[171] 2006	Israel-Palestine	7th-10th graders in Herzeliya, Israel	1,150	1/3 respondents were in the proximity during an attack and 40% knew someone who was injured (psychological proximity). Physical and psychological proximity to attacks were significantly associated with alcohol consumption, when controlling for PTSD and depressive symptoms.
114	Schiff et al,[172] 2007	Israel-Palestine	Jewish 10th and 11th graders in Haifa	960	Close physical exposure to armed conflict predicted higher levels of alcohol consumption, binge drinking among drinkers, and cannabis use.
115	Schiff et al,[173] 2012	Israel-Palestine	Jewish and Arab Israeli 7th-11th graders	4,151	The youth reported high rates of exposure to war events. Cumulative exposure to war events was significantly associated with alcohol and drug consumption and involvement in school violence.
116	Schlecht et al,[105] 2013	Uganda	Displaced Ugandan and Congolese refugee youth	133	Armed conflict resulted in breakdown of traditional community social structure and associated protective marriage practices. Displacement was also associated with social isolation and barriers in access to education. These social changes and challenges were associated with earlier sexual debut without involvement or knowledge of parents/caregivers, teen pregnancy, sexual exploitation of girls, transactional sex.
117	Shemyakina,[92] 2011	Tajikistan	Households with school age children across Tajikistan	6,160	Based on 2 surveys (one representative at national level and one at regional and urban/rural level). Children 8–15 years old in conflict-affected area were less likely to attend school. Damage to household dwelling negatively associated with the enrolment of girls. Nationally, men and women of school age during the war were less likely to complete nine grades of schooling compared to their pre-war counterparts.
118	Shuker,[174] 1985	not reported	Case report	1	7 year old child with multiple high-velocity wounds sustained from artillery shelling, including avulsion of 6cm of the left mandible. The mandible was stabilised with a K-wire, resulting in spontaneous regeneration of the entire osseous defect.
119	Skokic et al,[61] 2006	Former Yugoslavia	Newborns in Tuzla Canton 1992–2003	59,707	During the war: 20% fewer live births than pre-war and higher prevalence of premature delivery compared with pre- and post-war. Average birth weight of term newborns was 200 and 300 grams lower than pre- and post-war, respectively. Barriers to care during conflict evidenced by significantly fewer births attended by trained health providers and fewer prenatal visits.
120	Slim et al,[37] 1990	Lebanon	Patients with war-related abdominal injuries at a single facility	270	Children <16 years accounted for nearly 1/5 of abdominal trauma admissions. 70% of children had penetrating injuries from shrapnel, bullets, stab wounds, and blast explosions. The remaining 30% had blunt abdominal trauma. The children had 4.8% overall mortality; however patients transferred from other facilities had 55.6% mortality.
121	Soroush et al,[80] 2010	Iran-Iraq	Patients with landmine or UXO injury	3,713	Data from hospital records of 3713 patients in western and south-western Iran. 41.8% of patients with landmine or UXO injuries were <18 years old.
122	Spiegel et al,[175] 2011	Somalia	Refugee households in Dadaab camps	753	In a household survey of newly arrived refugees, 44 deaths reported, 29 (66%) were in children under 5.
123	Spinella et al,[40] 2008	Afghanistan and Iraq	Paediatric patients treated in 7 US combat facilities	1,305	Data from PASBA database. Report on paediatric trauma admissions December 2001—May 2007, or 7.1% of all trauma admissions. Children were severely injured at admission, had longer hospital stays, and accounted for 13% of trauma deaths. Age <6 years was associated with higher mortality rate (10.7% compared with 3.8% in children 6–17 years).
124	Stern et al,[176] 1995	Iraq	Case report	1	8 year old with aplastic anaemia, only known exposure was that his home was situated near to burning oil wells
125	Terzic et al,[43] 2001	Former Yugoslavia	Children treated at a tertiary care facility	94	Chart review of paediatric patients treated for war injuries. Most children were wounded by shelling and explosive devices, most commonly in the extremities. The most severe wounds were caused by shelling. 39.4% of children were permanently disabled and 3.3% died.
126	Trenholm et al,[114] 2013	DRC	Male former child soldiers	12	Ethnographic study. Boys reported abduction, forced recruitment, and poverty as reasons for entering the military. They describe beatings, forced marches, sleep deprivation, starvation, substance use, witnessing rape, and being forced to rape. The boys describe normalization of sexual violence, with rape considered a bounty of war and also as means to release anger or reap revenge.
127	Valente et al,[84] 2000	Angola	Polio cases in Angola	1,093	Description of a polio outbreak among displaced persons in Luanda. During a vaccination campaign in response to the outbreak, nearly 30% of districts could not be reached due to the conflict.
128	Van Herp et al,[64] 2003	DRC	Households in 5 conflict-affected regions of DRC	4,527	Threshold and emergency level U5 mortality rates at the front line, primarily due to malnutrition and infections. Context-specific variations in direct and indirect exposure to combat and barriers in access to acute and preventive health care.
129	Van Leent and Hopkins,[177] 1951	WWII	Refugee children in Australia	4,721	Sampled nearly 1/5 of refugee children, found low BCG vaccination rates. 36% were Mantoux positive, with a linear rise in Mantoux positivity with age: 3.9% positive at 1 year, up to 78% positive at 15 years.
130	Veale and Dona,[178] 2003	Rwanda	Street Children in Rwanda	290	87% came to the streets after the 1994 genocide. More than 3/4 had lost at least one parent, and 1/3 reported both parents were dead. 42% were homeless. The majority cited living on street due to changes in family structure, including loss of one or both parents, parent remarriage, becoming unaccompanied, being fostered in another family, or the closure of an unaccompanied children’s centre.
131	Verelst et al,[119] 2014	DRC	2nd and 3rd graders in Ituri district	1305	499 children (38.2%) reported having been victims of sexual violence. The risk of sexual violence increased with exposure to war-related violence, separation from family, being injured during the war, imprisonment, and association with armed groups.
132	Villamaria et al,[44] 2014	Afghanistan and Iraq	Paediatric vascular trauma patients treated in US combat facilities	155	Data from 2002–2011. The majority of vascular injuries in children were caused by blast injuries (58%), followed by bullets (37.4%) and falls (1.9%). Extremity injuries were more common while torso injuries were more lethal.
133	Vranković et al,[45] 1997	Former Yugoslavia	Case report	10	In 10 paediatric penetrating head injury patients, 4 were injured by bullets and 6 by shrapnel. Patients with shrapnel wounds had associated cerebral oedema. All gunshot wound patients survived; half of patients with shrapnel wounds died. 4 patients had moderate neurological deficits.
134	Weile et al,[55] 1990	Chile	Chilean refugee children in Denmark	58	Follow up of Cohn 1979. In child survivors of torture living as refugees in Denmark, there was an increase in the number of somatic symptoms and in the prevalence of symptoms over time. At follow up, 90% of children had one or more symptoms. Children who had continuous symptoms at the first study had significantly higher medication use. Number years lived in Denmark positively correlated to number of symptoms.
135	Wen et al,[70] 2000	Vietnam and Cambodia	Children with leukaemia	2,343	Pooled data from three studies shows increase in the risk for AML of the children of veterans who served in Vietnam or Cambodia (OR 1.7; 95% Cl: 1.0, 2.9). This risk was increased in fathers who had two or more tours of duty (OR = 5.0; 95CI: 1.0, 24.5).
136	Wilson et al,[38] 2013	Afghanistan	Paediatric trauma patients treated in a US combat facility	41	Data from facility trauma registry. 10% of trauma admissions were children, 71% were boys, and 59% were battle-related. More than 2/3 had penetrating injuries, most often from IEDs and landmines. 3/4 of injuries were severe, with AIS score ≥ 3, and 14.6% died.
137	Woods et al,[39] 2012	Afghanistan and Iraq	Paediatric trauma patients treated in British combat facilities	176	Data from JTTR. Half of paediatric (<16) trauma cases were less than eight years old. The most common mechanism of injury was explosive injury (59%), followed by gunshot wound (20.5%), and motor vehicle crash (8.5%). The mortality rate was 11%. 70% of mortality was due to explosive injury and 15% due to motor vehicle crashes. Half of deaths were due to head injuries.
**Search updates 25 April 2017**					
	**Author**	**Conflict zone**	**Population**	**Sample size**	**Summary of findings**
1	Bayarogullari et al,[179] 2016	Syria	Case report	2	Two children with complex shrapnel wounds resulting in vertebral artery pseudoaneurysms. One patient lost to follow up due to displacement.
2	Ceri et al,[180] 2016	Iraq	Yazidi refugee children in three camps	42	More than 2/3 of children reported sleep disturbances and >1/3 had somatic complaints
3	Charchuk et al,[58] 2016	DRC	Children in Bilobilo IDP camp and Mubi village	600	IDP camp residence predicted falciparum malaria in children (OR 2.6, 95% CI: 1.2–5.7). Bed net ownership and use were significantly lower for the children from the IDP camp compared to the children from the village.
4	Chi et al,[102] 2015	Burundi and Uganda	Health workers and women living in Burundi and Northern Uganda	115	Participants linked armed conflict with limited access to maternity and reproductive health services, poor quality of care, and increased neonatal morbidity and mortality. Conflict resulted in destruction and looting of facilities, targeted killing and abduction of health workers, and migration of health workers. Barriers in care resulted in increased use of traditional birth attendants. Girls 12–18 years from disadvantaged backgrounds were noted to be at high risk for sexual exploitation, unintended pregnancy, and subsequent health complications.
5	Cook et al,[49] 2015	Myanmar	Karen refugees in Minnesota, USA	179	Children described witnessing and being subjected to war-related violence, including witnessing parents and community members being tortured and killed by soldiers, repeated and forced displacement, being beaten, child labour, and being forced to join the military.
6	Denov and Lakor,[115] 2017	Uganda	Children born to mothers abducted by the Lord's Resistance Army	60	Children report witnessing combat and mass executions, seeing injured children and dead bodies. They reported being orphaned, being abandoned by parents, going without food or water, and lack of access to health care and education. Stigma and social exclusion from parents, families and communities led some children to describe life during war as preferable because they were loved, had a caring father present, and a sense of family.
7	Duque,[181] 2017	Colombia	Children in Colombia	13,344	Violence exposure in first trimester of gestation and in early childhood was associated with decline in math reasoning, general knowledge, and Peabody Picture Vocabulary Test scores.
8	Guha-Sapir et al,[47] 2015	Syria	Civilian violent deaths in Syria 2011–2015	78,769	Children accounted for >16% of civilian deaths in non-state controlled areas and >23% of civilian deaths in government-controlled areas. The risk of death from different combat activities varied by location, however, children in all areas were more likely than men to die from air bombardments, shells, ground level explosives, and chemical weapons. 852 children were killed by execution, including execution after torture.
9	Hemat et al,[182] 2017	Afghanistan	Trauma patients at single facility	35,647	Paediatric trauma patients at Kunduz Trauma Centre Jan 2014- June 2015: Children accounted for 50% of patients registered in the emergency department and 41% of operated patients.
10	Khamaysi et al,[183] 2015	Syria	Case report	5	Report on 5 patients with traumatic bile leaks from war injuries, including two children.
11	Klimo et al,[30] 2015	Iraq and Afghanistan	Paediatric neurosurgical patients at US military combat facilities	647	Data from JTTR. Review of neurosurgical cases 2004–2012. 76% of patients were boys, with a median age of 8 years. 60% of patients were under 9 years of age. 61% had penetrating head injuries. Most commonly mechanism of injury was IED explosion, blast, gunshot wound, and mortar. In-hospital mortality was 24%.
12	Lindskog,[125] 2016	DRC	Infants born in DRC	53,768	Uses DHS data to analyse infant mortality among 15,103 mothers and 53,768 children. Infant mortality was higher during the war years compared with pre-war and post-war. There was a linear relationship between post-neonatal infant mortality and the number of conflict events
13	Nnadi et al,[83] 2017	Nigeria	Polio cases in Borno	4	There were 4 reported cases of Polio in the region. Two infected children were unvaccinated, and two were partially vaccinated. In the two years preceding the case report, 50% of settlements in Borno were inaccessible to public health programmes.
14	Rabenhorst et al,[96] 2015	Afghanistan and Iraq	US Air Force personnel deployed >30 days	99,679	There were no overall changes in substantiated child maltreatment (CM) rates post-deployment compared to pre-deployment, however rates of child injury increased post-deployment (RR1.6, 95% CI: 1.31, 2.01), as well as rates of moderate and severe maltreatment (RR 1.9, 95% CI: 1.34, 2.80) and CM involving alcohol use (RR 1.5, 95% CI: 1.11, 2.15).
15	Rouhani et al,[118] 2015	DRC	Women raising children born from sexual violence	757	More than a third report stigma toward their child from the community and 2/3 reported often seeing their assailant and/or remembering the sexual assault when looking at the child. Stigma and maternal mental health disorder was associated with negative parenting attitudes. Family and community acceptance were associated with adaptive parenting attitudes.
16	Stark et al,[65] 2015	Uganda	Congolese and Somali refugees in Kampala	>215	175 In-depth interviews, 40 key-informant interviews and 51 focus group discussions. Children reported discrimination in schools and teachers encouraging xenophobia Conversely, some reported reduced school fees and accommodations made for prayer. Children reported social marginalization in the community, barriers in access to sanitation, assault, and lack of access to health care and legal and protective services.
17	Sullivan et al,[98] 2015	multiple	Californian children in public civilian schools, grades 7, 9, and 11	688,713	Military-connected secondary school students reported higher levels of physical violence (OR 1.47, 95% CI: 1.43–1.50) and nonphysical harassment (OR 1.42, 95%CI: 1.38–1.45). They had twice the odds of carrying a gun to school (OR 2.2, 95% CI: 2.10–2.30), and nearly twice the odds of carrying a knife or other weapon to school (OR 1.81 (95% CI: 1.75–1.88).
18	Weishut,[56] 2015	Israel-Palestine	Palestinian victims of sexual torture by Israeli authorities	77	Data on 77 cases from Public Committee Against Torture in Israel database. 15% of victims were minors. They describe beating of the genitals, forced confession, threats of rape, and threats of sexual violence against family members.

### Direct health effects

Children exposed to armed conflict suffer a broad range of injuries and illness that can be directly attributed to conflict (Fig 3). One third (N = 52) of included studies describe a range of physical injuries affecting all organ systems, broadly classified as penetrating injuries, blunt trauma, crush injuries and burns. Injuries were attributed to shelling, explosions, collapsing buildings, gunshots, and motor vehicle crashes.

Among injured children who reach health facilities, penetrating injuries are most common.[25–39] Penetrating head injury is the most frequent form of head injury among children treated in military combat facilities, accounting for 60–75% of all head injuries and carrying the highest mortality risk.[28, 30, 32] This pattern of head trauma differs markedly from that observed in peaceful settings, where blunt head trauma predominates. It is important to note that the admission criteria for combat support hospitals, access to military facility care, and care seeking behaviours of people living in combat zones are likely to influence the findings in military studies; Spinella et al documented that a child with a severe head injury had sought care at five other hospitals before presenting to a military facility.[40]

Reported mortality from trauma ranges from 2.6–18%,[33–44] and as high as 24% in neurosurgical patients.[30–32, 45] Younger trauma patients bear a significantly higher burden of mortality when compared with older children and adults.[40, 41] No studies provided data on mortality after transfer or discharge from health facilities.

While numerous case studies report children with traumatic amputations, only one study examined the prevalence of disability among war-injured children. This single facility retrospective chart review of 94 children with war-related injuries sustained during the wars in Croatia and Bosnian and Herzegovina found that nearly 40% of the children suffered permanent disability.[43] No studies describe the incidence or prevalence of childhood disability associated with armed conflict or its long term effects on health, development, or life opportunities.

Two studies document the effects of chemical or biological weapons on children. Momeni and colleagues describe the clinical manifestations of mustard gas exposure in a group of children during the Iran-Iraq war.[46] The paper highlights the difference in presentation when compared with adults, including earlier onset of symptoms, more frequent pulmonary and gastrointestinal symptoms, and predominant face and neck symptoms. Guha-Sapir and colleagues found that children in the ongoing conflict in Syria are twice as likely to die from chemical weapon attacks as adults (OR 2.11, 95% CI: 1.69–2.63).[47]

Studies documenting the torture of children report a variety of torture methods that children experienced and/or witnessed. These children suffer physical injuries, a variety of somatic complaints, enuresis, constipation, sleep disorders, and psychological disorders.[47–56] A follow-up study on children who were tortured or whose parents were tortured in Chile found that both the prevalence and frequency of somatic symptoms increased over time after resettlement in Denmark.[55]

### Indirect health effects

#### Diseases

Exposure to armed conflict is associated with a higher burden of infectious, communicable, and noncommunicable diseases in children. A global study of disability-adjusted life years (DALYs) associated with civil war found significantly reduced DALYs in children under 14 years for all disease categories, with the most severe reductions in the under 5 (U5) age group.[57] Diseases such as malaria,[58] diarrhoea, acute respiratory infections, and fever[59] are more common and carry higher mortality.[60] A nationwide cross-sectional study in Iraq after the first Persian Gulf War found that age-adjusted mortality due to diarrhoea rose from 2.1 pre-war to 11.9 per 1000 person-years after onset of war.[60] Pregnancy and birth in conflict zones are also higher risk. A study in Tuzla Canton, Bosnia, found significantly fewer live births, increased preterm delivery, and low birth weight during wartime, which normalised again after the war.[61] Displacement due to conflict is associated with crowding, limited access to water and sanitation, and increased risk of infectious and communicable diseases.[62–65]

#### Environmental exposures

Studies on the effects of combat-related environmental exposures have identified an increased prevalence of birth defects and cancer, as well as ongoing risk of injury due to unexploded ordnance (UXO). The incidence of structural heart defects in Kuwaiti infants rose significantly after the first Persian Gulf War to levels exceeding the international incidence.[66] While the mechanism for this jump in prevalence cannot be identified by the study design, the authors suggest that war-related environmental pollution may play a role. This hypothesis is supported by a large study of birth defects in children of US Gulf War Veterans using active case ascertainment.[67] Conversely, an earlier review of military health records suggests no increased incidence of birth defects among the infants of US Gulf War Veterans.[68]

A study in Iraq found a significantly increased rate of leukaemia, particularly amongst younger children.[69] Similarly, Wen and colleagues found significantly increased risk of AML in children whose fathers reported having served in Vietnam or Cambodia.[70] AML risk was further elevated if fathers reported two or more tours.

Landmines and unexploded ordinance (UXO) remain a significant risk for children during conflict and long after combat has ended.[71–80] The burden of injury due to UXO varies by conflict, however children account for approximately half (42–55%) of injuries from UXO in Afghanistan, Nepal, Eritrea, and Iran.[71, 73, 74, 77, 80] The pattern of injuries in children is similar across settings; children more often suffer upper body injuries sustained while playing, going to school, or tending animals.[71–74, 78, 79]

#### Access to basic needs

Whilst the burden of disease increases due to conflict, access to health care becomes more difficult. Children in areas affected by conflict are less likely to receive vaccinations [81, 82] and conflicts may contribute to outbreaks of vaccine preventable diseases such as the polio outbreaks during the conflicts in Nigeria and Angola [83, 84] and a rubella outbreak in Bosnia and Herzegovina after hostilities had ended.[85] Combat may also hamper vaccination campaigns during disease outbreaks.[84] Several studies have reported that pregnant women displaced due to war were less likely to receive prenatal care or deliver with the assistance of a skilled birth attendant.[61, 82, 86, 87] Studies from the former Yugoslavia describe drastic reductions in the number of health personnel [88] and conditions where children with chronic conditions had less frequent access to medical care and experienced worsening of their condition.[89]

Conflict also prevents children from going to school and lowers their overall educational attainment. A large time-series cross-sectional study examining the impact of war in sub-Saharan Africa (1950–2010) found that armed conflict significantly reduced school attendance for both boys and girls, and with an inverse correlation between military expenditure and school attendance.[90] Similar findings have also been described in Palestine,[91] Tajikistan[92] and Colombia.[93] The Colombian study findings suggested this may have a long-term effect—adults in areas affected by conflict had lower educational outcomes.

#### Risk of abuse, neglect, and exposure to secondary violence

Children whose caregivers have been exposed to armed conflict are at increased risk for child abuse and neglect. Studies from Timor-l’Este, Uganda, and in Syrian refugees in Lebanon found that caregivers who suffer from stress or have a mental health disorder related to their exposure to armed conflict have higher rates of child-reported and caregiver-reported child abuse.[87, 94, 95] The Ugandan study findings suggest that war exposure and subsequent trauma are mediating factors for violence against children. Studies among U.S. military personnel have documented increased rates of physical abuse and neglect in the children of veterans, both during the period of deployment and after return.[96, 97] A large study of Californian children in civilian public schools found that the children of US military personnel have higher rates of experiencing violence in school and are more likely to carry a weapon.[98]

#### Social changes

Important changes in societal structure, norms, and roles take place in populations affected by conflict. Children may assume adult responsibilities, including providing for their families and caring for ill or disabled parents.[99–101] Several studies described changes in sexual behaviour, with earlier sexual debut and child marriage.[102–105] Displacement and separation from family may place children at increased risk for exploitation, high risk sexual behaviour, sexually-transmitted illness, and teen pregnancy.[104] Two studies from Uganda describe particular barriers in access to information and health care for adolescents that further compound their health risks.[104, 105]

#### Toxic stress and child development

While numerous studies document the multiple kinds of war-related violence that children witness, no studies examine the effect of these exposures on child motor and psychosocial development. Two studies examined the influence of war-related trauma on children’s stress physiology. Feldman et al. found that war-exposed children had altered cortisol and salivary amylase response to stress.[106] The same study also found that children’s baseline cortisol levels were independently related to maternal baseline cortisol, mother-child relationship, and maternal mental health. Similarly, the Helsinki Birth Cohort Study described altered stress physiology in children who were separated from their parents for a period during WWII.[107] Two other studies on the same birth cohort found that separated girls had significantly earlier onset of menarche than non-separated girls, and that children separated from their parents in early childhood had lower scores on intelligence testing, respectively.[108, 109]

#### Children associated with armed forces or armed groups (“child soldiers”)

Studies from Sierra Leone, the Democratic Republic of the Congo (DRC), and El Salvador document the extreme kinds of violence that children experience during association with armed forces or groups, including being forced to watch and take part in killing, cannibalism, rape, child marriage, and sexual slavery.[52, 99, 110–114] Three studies also describe the process of indoctrinating children and methods used to control and isolate them. The children in these studies describe regular physical and psychological abuse, torture, and the normalisation of violence.[52, 113, 115] In the words of one child: “After some time, [the violence] became part of me”.[113]

Several papers describe unwanted teenage pregnancies, gynaecological problems, and sexually transmitted illnesses among child soldiers.[99, 111, 112] A case report on three former child soldiers in the DRC describes a combined total of 403 medical complaints and 275 physical findings in multiple organ systems.[52] The sheer number and diversity of complaints and somatic findings in these three youth provides insight into the extensive harm wrought by association with armed groups.

#### Sexual violence

The witnessing, experience, and perpetration of sexual violence and sexual exploitation are documented by numerous papers in this review, nine of which dealt exclusively with these issues.[56, 100, 104, 105, 115–119] All included studies of children associated with armed groups document sexual violence of and by these children. However, the studies that focus exclusively on sexual violence and exploitation, suggest the problem is far more widespread. These studies describe children being: a) threatened with rape, b) raped, including gang rape, c) forced to watch rapes, including rapes of family members, and d) forced to rape others and engage in sexual slavery. They also document “survival-”, or transactional sex to obtain basic needs, and early marriage. Documented physical findings include traumatic genital injuries, vesicovaginal fistulae, and pregnancy. Two studies describe the experiences of children conceived through sexual violence, including social isolation and exposure to violence and threats from within their communities.[100, 118] A survey of women raising children conceived through conflict-associated rape reported that 66.1% often saw their assailant and/or remembered the sexual assault when they looked at their child.[118]

### Mortality

More than one-third (n = 64) of studies included mortality as a main outcome. The heterogeneity of design and lack of denominators in much of the reported data preclude the pooling of data. However, numerous studies report significant increases in infant and child mortality during periods of armed conflict, in comparison to peacetime or when compared with peaceful parts of the same country.[21, 60, 82, 120–127] Most of these studies report on under 5 years (U5) mortality.

A pooled study of 37 datasets from 1985–2001 found that the relative risk of dying during periods of conflict was higher for older children (≥ 5 years) when compared to those U5.[122] In some countries, U5 mortality actually decreased during the period of conflict. The absolute U5 mortality remained higher than the ≥ 5 years mortality independent of the presence of conflict, suggesting that additional factors unrelated to conflict were contributing to U5 mortality in these populations.[122] This study suggests that there are nuanced and context-specific factors that lead to conflict-associated mortality during different periods of childhood.

Further examples in support of the importance of context on mortality outcomes is seen in studies by Ascherio and Nielsen that found maternal education is protective against child mortality in conflict settings.[60, 126] Avogo et al. found displacement of children due to war carries a higher mortality risk than displacement for other reasons, with the highest risk of death during the year after migration.[86] The study suggests that forced migration due to war exposes children to health risks after resettlement to which other migrant children are less vulnerable. Mortality studies by Hicks[53] and Guha-Sapir[47] document the direct deaths of children due to combat activities in Iraq and Syria, respectively. In addition to deaths from bombs and gunshot wounds, both studies document the torture and execution of children.

### Geographic distribution of the literature

There is a notable trend in the geography of published studies. During the period 1946–2016, there were 280 distinct armed conflicts.[4] Papers providing data on child physical health and development are focused on a few specific conflicts and on particular regions of the world. There is a remarkable dearth of data on major past conflicts, such as the Korean War and the Chinese Civil War, and more recently the conflicts in Yemen, Myanmar, Guatemala, Mexico, the Central African Republic, and Pakistan. There is also a notable lack of data on protracted conflicts, such as those in Kashmir and in Xinjiang province, China.

**Fig 4:**
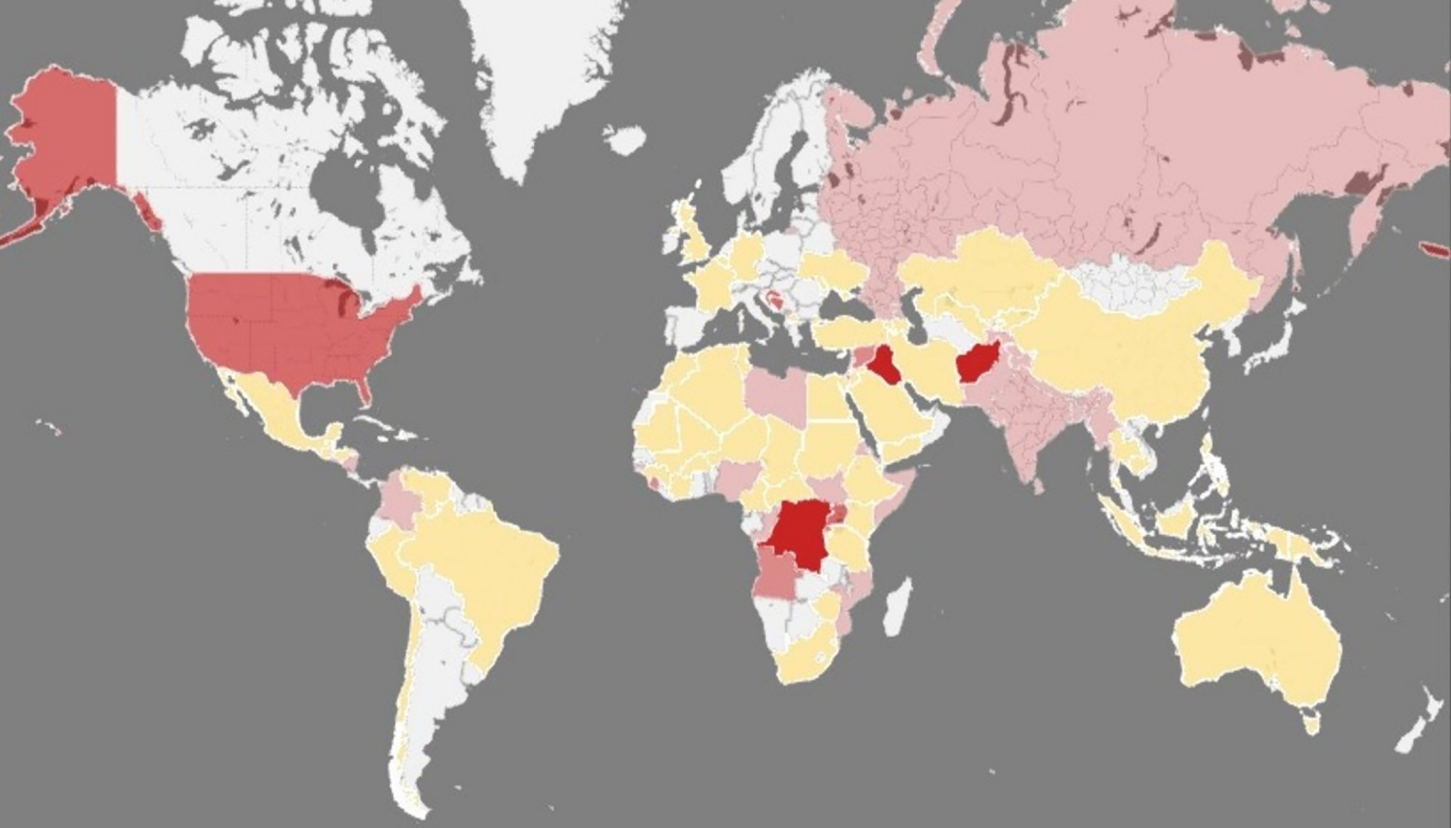
The number of published studies on child health in countries with documented armed conflict 1990–2016.

**Fig 5:**
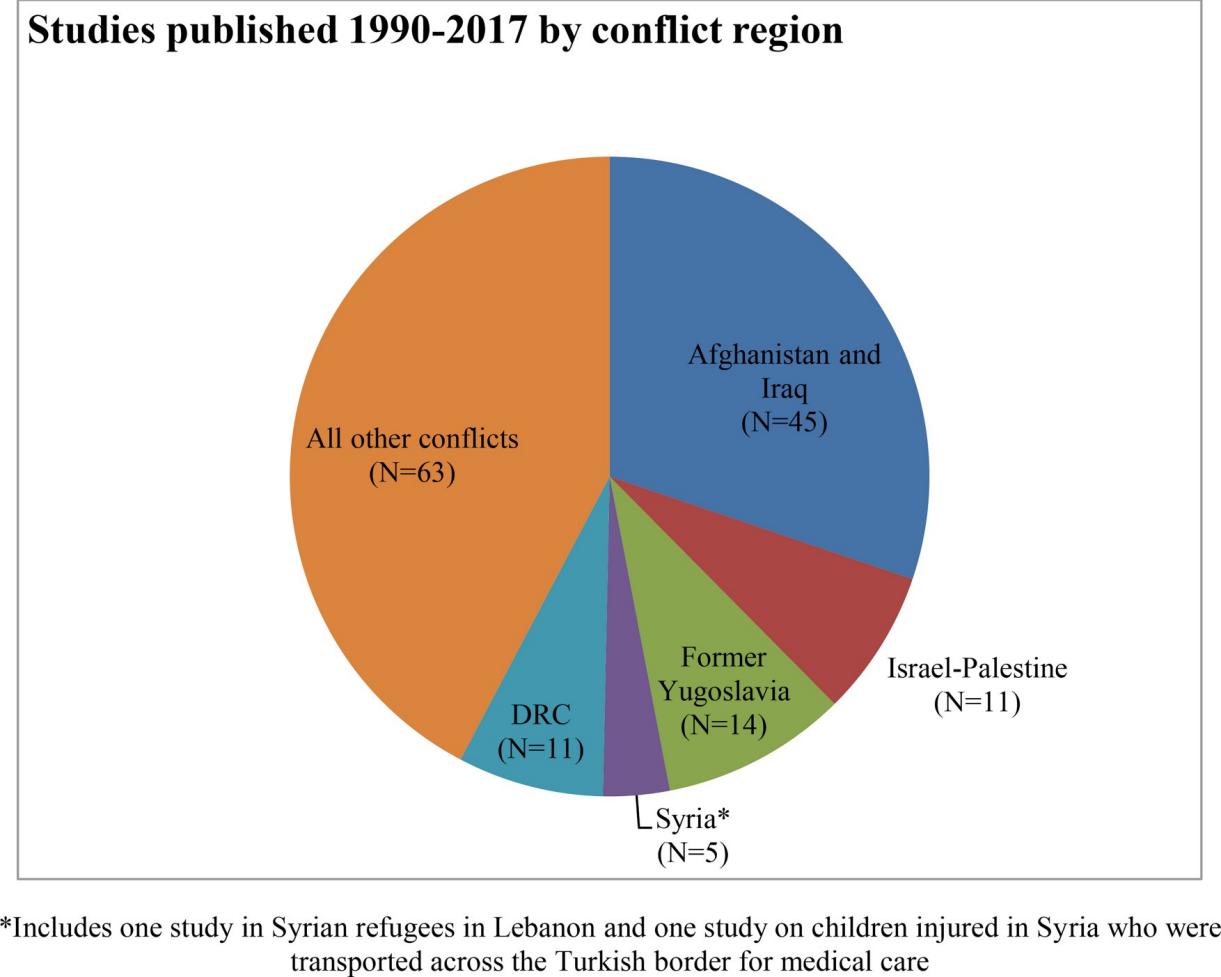
Studies published 1990–2017 by conflict region (number of studies in parenthesis).

Figs 4 and 5 illustrate the publication trend from 1990–2016. During this period, there were 165 armed conflicts across all regions of the globe.[22] Child health is studied in only 30 of these conflicts, and 60% of published studies focus on just six conflict zones. Closer examination of geographical reporting patterns reveals a conflict-specific focus on exposures and outcomes. For example, seven of the nine studies examining sexual violence against children were conducted in the DRC and Uganda.[104, 105, 115–119] All four studies from Sierra Leone focus on child soldiers. Of the 45 studies examining child health in Afghanistan and Iraq, half are case studies and chart reviews from military combat facilities.

## Discussion

This is the first systematic review of the global impact of armed conflict on child physical health and development. The review’s findings reveal the breadth of the problem, its pervasive and sustained impact on child health, and important nuances in the way conflict affects child health. Further, it describes how these effects differ based on the direct and indirect nature of the impact, characteristics of the individual conflict (e.g., types of combat, weapons, displacement, relief efforts), and contextual factors associated with the geographic location of the conflicts. The broad view given by the available evidence is quite disturbing and therefore warrants reporting in this first paper on our systematic review. Further analysis of subsets of the data will be presented in future publications.

It is important to reiterate that this review deliberately excluded papers reporting exclusively on child health outcomes that are known to carry high risk in conflict settings, including nutrition, perinatal mortality, infant feeding, and immunization coverage. Most papers that reported on perinatal mortality, birth weight, and infant feeding also reported on other child health outcomes, and were thus included in this review. However, the relative paucity of papers in our review providing data on these topics suggests that the evidence for the effect of armed conflict on maternal and newborn health is lacking. This finding is consistent with other recent reviews on the subject.[128, 129] Additionally, there is a large literature on the effects of forced displacement on child mental health,[19, 20] much of which was not identified by the searches. Finally, studies that were published as books were not included in the review; this is likely to have precluded the examination of a number studies, particularly those undertaken before 1990.

The grey literature database searches retrieved only 382 citations, none of which met inclusion criteria. This review is therefore limited to the peer-reviewed literature; this is an important limitation because traditional public health research in conflict settings is limited by security concerns, population movements, the destruction of public health infrastructure, and the disruption of routine data collection.[13] Furthermore, most of the studies rely on health facility data and are thus likely have selection bias, as they report on those patients who were able to access care in a facility where sufficiently complete records were maintained and were accessible to the researchers. Much of the available information about the health of populations living outside of refugee or IDP camps in areas of active conflict is not reported in the peer-reviewed literature and is therefore missing from this review. Among studies providing data from areas experiencing active conflict, the majority were retrospective chart reviews. Of the 11 population-based studies in settings with active conflict, 10 used a cross-sectional and/or qualitative design. [60, 64, 81, 106, 126, 130–134] While there were no marked differences in the findings from these studies compared with studies in refugees in neighbouring regions, the design of the studies make it difficult to draw further conclusions.

Through its pervasive harmful effects on children, armed conflict is a negative social determinant of child health. Numerous studies have documented that adversity during childhood can alter the architecture of the brain and neuroendocrine function, leading to alterations in learning, behaviour, and physiology, in turn predisposing the developing child to maladaptive behaviours and ill health throughout the life course.[135] The findings on stress physiology reported in this review are in keeping with previous studies on toxic stress and childhood adversity, and therefore have significant implications for generations of children growing up amid armed conflict.

For each individual child exposed to armed conflict, the way in which this exposure affects the child’s health is likely to be determined by a number of factors including genetic predisposition, physical health, mental health, development, behaviour, caregiver physical and mental health, forced displacement, and social arrangements. The influence of caregiver mental health on the physical and mental health of conflict-affected[9] and forcibly displaced children[8] is well-described. The findings of Feldman et al[106] suggest that in war-affected children, caregiver attachment and mental health may play a mediating role, and is therefore a potential area for intervention to mitigate the effects of armed conflict on children.

Numerous studies in this review report on physical health, mental health, and social conditions in the same population, suggesting they are interrelated. Other studies describe alterations in social arrangements due to conflict, such as disruption of communities and barriers in access to education, which affect child and adolescent health and wellbeing. The relationship between physical health, mental health, and social conditions in conflict-affected children is an area in need of further research, and that may provide insight into aggravating factors, mitigating factors, and ways to promote good health and resilience.

The overlap of different typologies of violence against children in the context of armed conflict is an important finding that merits further attention. In addition to risks for being injured, tortured, and/or a witness to or participant in combat, children with direct and indirect exposure to conflict are also at increased risk for other forms of violence, including abuse and neglect,[87, 94–97] community and school violence,[98] and domestic violence.[94, 95] Furthermore, Nelson et al describe an increase in civilian-perpetrated rape of children during a period of conflict in the DRC.[117] While these forms of violence against children are extensively described in grey literature reports, the association of these forms of violence with armed conflict points to an important need for child protection after the exposure to conflict has ended.

**Fig 6:**
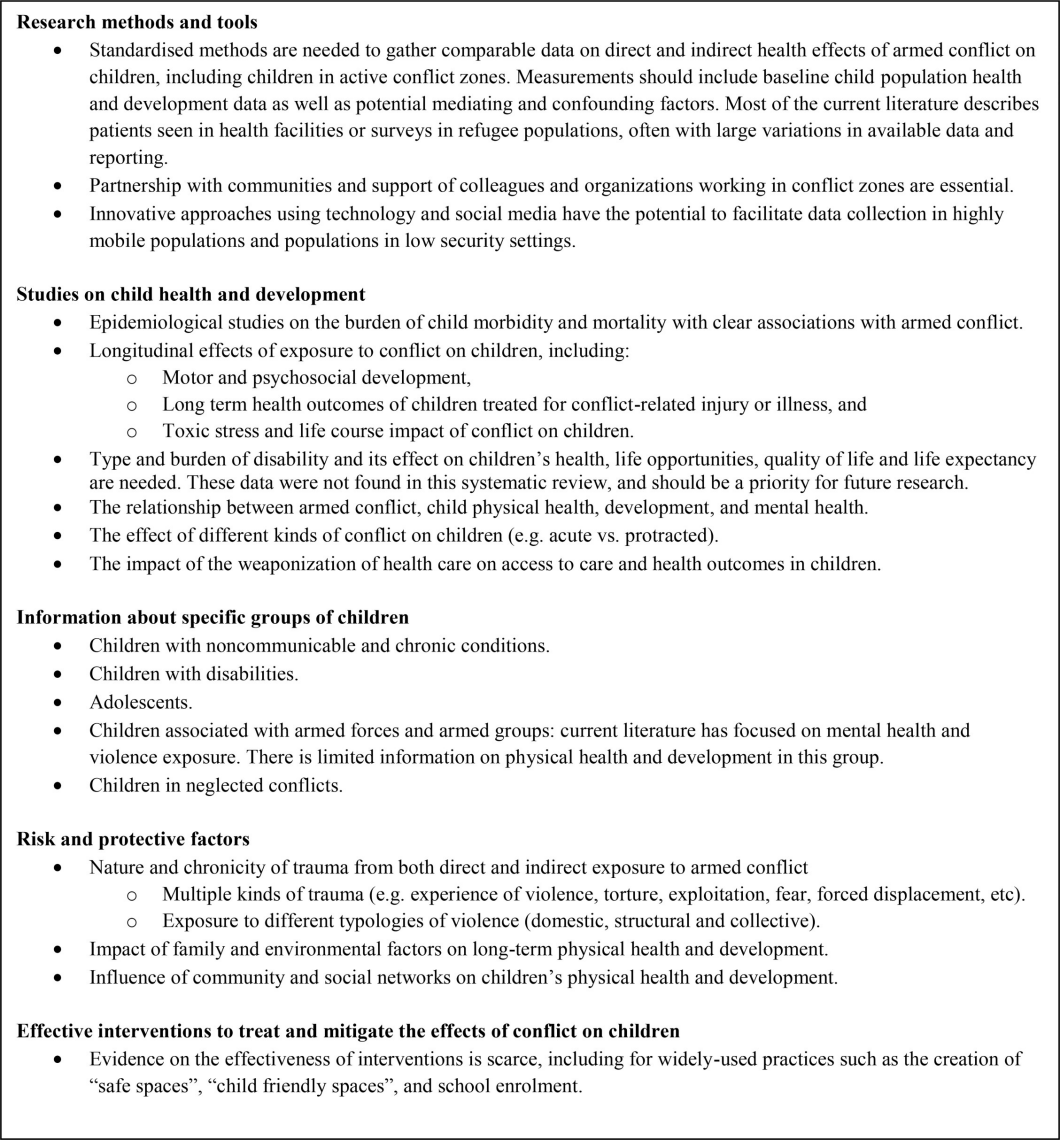
Recommendations for future research.

There is a compelling need for further research to improve our understanding of the medium and long term effects of the exposure to armed conflict as a form of violence against children. Areas in need of further study include global child development, children with chronic diseases, children with disabilities, and adolescent health. Of particular importance is the need to improve our understanding of the relationship between physical and mental health and social conditions in conflict-affected children and factors that protect and mitigate the harmful effects of armed conflict (Fig 6). In addition to informing the development of evidence-based interventions to treat and mitigate the harmful effects of conflict on children, such knowledge would improve our understanding of how to advance the health and well-being of adults who have experienced armed conflict and other adversities during childhood and adolescence. Such information would be of great use for peace research and conflict resolution.

The studies included in this review demonstrate that it is possible to study the ways that armed conflict affects child health and development using both quantitative and qualitative methods. However, the vast majority of the literature is descriptive because traditional epidemiological research is difficult to undertake in settings with insecurity and disrupted health systems. A study published after completion of this systematic review brings attention to a potential way to improve the study of conflict and child health and highlights an important role for humanitarian organizations working in settings where routine data is no longer available. Meiqari et al[136] describe paediatric data collected by Médecins Sans Frontières (MSF) in Tal-Abyad and Kobani, Syria. In spite of challenges, which included population movements and abrupt closing and opening of clinical services due to the shifting front lines of the conflict, the authors describe the epidemiology of a large cohort of children who received care in MSF facilities for periods between 2013–2016, including 27,742 children U5 seen in outpatient clinics, 4672 children under 18 years admitted for inpatient treatment, and a measles epidemic response. Meiqari and colleagues provide important data about child health epidemiology in the study region, as well as information on care-seeking behaviours in a conflict zone notorious for attacks on health care facilities.[137] By supporting colleagues working in conflict zones and developing epidemiological methods to better study health in low-security settings, we can improve the ability to conduct research that can meaningfully improve the care and outcomes of children affected by armed conflict.

## Conclusion

This systematic review documents the pervasive effect of armed conflict as a form of violence against children and negative social determinant of child health. The studies serve as a record of the continuing occurrence of the six grave violations of children’s rights, which include the killing and maiming of children; recruitment or use of children as soldiers; sexual violence against children; abduction of children; attacks against schools or hospitals; and the denial of humanitarian access for children. There is an urgent need to improve our research on the mechanisms by which conflict affects child health and development and the relationship between physical health, mental health, and social conditions. Priority should be given to studies on child development, the long term effects of exposure to conflict, and protective and mitigating factors against the harmful effects of conflict on children. Collaboration with partners across sectors and incorporating a child rights perspective into research can improve both our understanding of the effect of conflict on child health as well as our response to their needs.

## Supporting information

S1 Checklist PRISMA checklist. (DOC)
