# Correction: Effects of armed conflict on child health and development: A systematic review

**DOI:** 10.1371/journal.pone.0212393

**Published:** 2019-02-11

**Authors:** The PLOS ONE Staff

The legend and source are missing from Fig 4. Please see the correct Fig 4 here. The publisher apologizes for the error.

**Fig 4:**
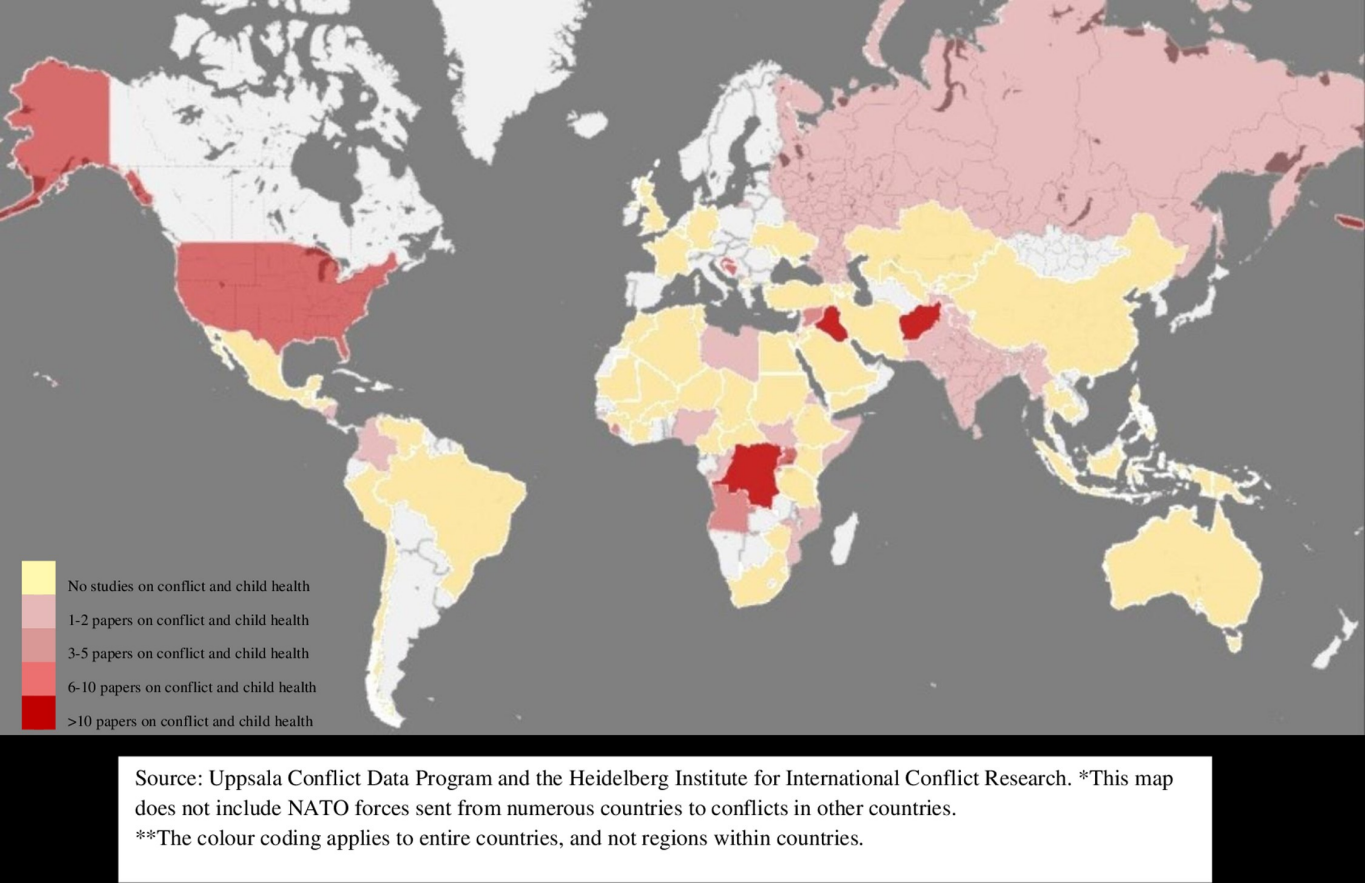
The number of published studies on child health in countries with documented armed conflict 1990–2016.
